# Editorial: One health, pandemic prevention, climate health education: the importance of interprofessional education pedagogy

**DOI:** 10.3389/fmed.2026.1976243

**Published:** 2026-09-24

**Authors:** Rohini Roopnarine, Brian Sick, Anuja Riles

**Affiliations:** 1 St George’s University, Saint George's, Grenada; 2 International Institute of Geopsychiatry, Bern, Switzerland; 3 Center for Interprofessional Health, University of Minnesota, Minneapolis, MN, United States; 4 University of Colorado Anschutz School of Medicine at Colorado State University, Fort Collins, CO, United States

**Keywords:** accreditation, authentic learning, interprofessional education, one health, planetary health

## Introduction

Many articles describe the overlap of One Health and interprofessional education (IPE), with One Health providing the “why,” the need for collaboration across the professions to address complex health problems, and IPE providing the pedagogical platform for “how” these problems can be addressed. The rationale for the choice of topic for this Research Topic was the renewed recognition of the One Health approach in pandemic prevention, following the COVID pandemic. The primary objective of this collection was to catalyze academic dialogue regarding the design of curricula that embed the One Health framework within multiple healthcare disciplines, including medical, veterinary, and public health education.

### Major contributions of the collection

#### A. Learning through authentic experience

A consistent message across the articles included in this topic is that students learn One Health most meaningfully when education is grounded in authentic problems rather than limited to theoretical knowledge. Habermehl et al. provide a strong example, putting students in the role of problem solvers as they developed a framework to address a real-life, community-grounded One Health challenge while considering the interconnected human, animal, and environmental dimensions of the challenge. Similarly, Yapi et al.'s work on brucellosis began with an issue affecting the students' own community. Rather than learning about brucellosis only as a disease, students identified a need for greater awareness among health professionals and developed an intervention to address that need. Cariddi et al. also used real-world cases, including environmental justice concerns and PFAS “forever chemicals,” to make planetary health education current and consequential. These experiences allow students to grapple with uncertainty, competing priorities, and the social dimensions of health, all experiences that cannot be fully reproduced through lectures or traditional learning.

Authentic experience also provides a meaningful foundation for IPE, particularly when different professions come together because of a shared community or educational need. Hobusch et al. illustrate this through One Health doctoral students and future teachers co-designing a curriculum, bringing together different professional perspectives to a common challenge. This model demonstrates that IPE need not be an additional activity added to an already crowded curriculum but can be embedded in work that benefits participating professions and the communities they serve. Habermehl et al.'s One Health Trail required students to examine a challenge from multiple perspectives rather than through the lens of a single discipline. Parsons and Watson's use of real-world social entrepreneurship cases allowed learners to consider how different specialties and domains contribute to addressing social problems. Collectively, these examples suggest that IPE is most meaningful when embedded in authentic challenges that require students to apply their disciplinary perspectives while learning to work with others. Communities can help identify the problems that need to be addressed, while educational programs can provide opportunities for students from different professions to develop the knowledge and collaborative skills needed to respond to those problems.

#### B. Broadening One Health Scope and Reach

It is understood that One Health involves the intersection of animal, human, and environmental health. Although some equate environmental health with climate change, Potter reminds us that the challenge is much broader. We are in the midst of a planetary crisis in which we have exceeded seven of the nine planetary boundaries which support life on Earth, of which only one of these boundaries is climate change. The solutions to these challenges require us to go beyond interprofessional collaboration among the health professions and towards transdisciplinary planetary health education in which disciplines such as engineering, agriculture, and the arts learn and work alongside health professions. Ly and colleagues provide an example of how to foster this collaboration. Using data from the Planetary Health Report Card, faculty and leaders at the University of New England created an interprofessional education event about the effects of pharmaceutical waste on planetary health, showing us how a planetary challenge can bring learners together for a meaningful and engaging educational activity. The article by Yusuf et al. provides a fresh, whole-country look at the awareness and attitudes of health professionals towards One Health in Somaliland. Most had heard of One Health but had not received any formal training and did not understand its implications. The article provides previously undescribed baseline data for a population and illustrates the importance of continued work in expanding the reach of awareness campaigns around One Health.

#### C. Challenges and Solutions

Berrian et al. and Gruszynski et al. remind us that integrating One Health and IPE is not without challenges. These include a shortage of faculty with One Health expertise, unclear career pathways for graduates of One Health programs, difficulty navigating tuition and faculty effort for cross-institutional coursework, and differences in educational approaches across the professions. Another challenge is the varying accreditation requirements across the professions. For example, in McMillan et al., the authors explain that while the Liaison Committee on Medical Education (LCME), which accredits medical schools, does not have explicit standards for how One Health can be integrated into medical education, several pedagogical approaches used in teaching One Health curricula, such as problem-solving skills, critical judgment, and systems-based practice, align with LCME standards. Both McMillan et al. and Cariddi et al. provide examples of how they used these pedagogical approaches, such as problem-based cases, workshops, and virtual panels, to educate students on One Health topics. Gruszynski et al. identified strategies to sustain these efforts including institutional support through protected faculty time, funding, coordination, and supportive policies; integration of One Health into clinical practice such as asking about pets in human healthcare practices and discussing the impact of environmental health on the health of animals and humans; and research that moves beyond measuring One Health knowledge and into changes in attitudes, behaviors, and ultimately clinical practice. An underappreciated challenge is how healthcare professionals describe their professional identity given their unclear understanding of One Health and IPE. Topka and Hancock captured the narratives of veterinary students who were confused when they tried to reconcile their identity as a veterinarian as well as their identities as an interprofessional team member and a One Health collaborator. The emergence of dual or triple identities in One Health and IPE is a challenge warranting further research.

## Conclusion

This collection illustrates a field transitioning from a call to action for One Health to operationalizing it through educational innovation and through the lens of IPE. By integrating interprofessional learning, authentic community engagement, and a broader scope and reach of One Health, these articles collectively offer practical approaches for preparing the next generation of health professionals to address interconnected global health challenges, including the effects of climate change on human, animal, and planetary health. The insights offered by these published contributions illustrate the significant work already being done in the design and implementation of One Health and IPE programs and curricula. They also demonstrate the need for continued engagement and collaboration, particularly as it pertains to developing accreditation standards for interprofessional programs as a platform for One Health and the sustainability of our planet.

